# Whey Protein Isolate Hydrogels Containing Cannabidiol Support the Proliferation of Pre-Osteoblasts

**DOI:** 10.3390/gels11060418

**Published:** 2025-05-30

**Authors:** Daniel K. Baines, Varvara Platania, Nikoleta N. Tavernaraki, Karen Wright, Maria Chatzinikolaidou, Timothy E. L. Douglas

**Affiliations:** 1 School of Engineering, Lancaster University, Gillow Avenue, Lancaster LA1 4YW, UK; d.baines3@lancaster.ac.uk; 2 Department of Biomedical and Life Sciences, Biomedical and Life Sciences Lancaster University, Gillow Avenue, Lancaster LA1 4YW, UK; karen.wright@lancaster.ac.uk; 3 Department of Materials Science and Engineering, University of Crete, 71003 Heraklion, Greece; plataniavarvara@yahoo.com (V.P.); ntav@materials.uoc.gr (N.N.T.); mchatzin@materials.uoc.gr (M.C.); 4 Institute of Electronic Structure and Laser, Foundation for Research and Technology Hellas, 70013 Heraklion, Greece

**Keywords:** whey protein, hydrogel, cannabinoids, hydrophobic, swelling, drug delivery, biocompatibility, bone tissue engineering

## Abstract

Bone-associated pathologies are major contributors to chronic pathology statistics. Current gold standard treatments present limitations such as the ability to act as scaffolds whilst effectively delivering medications to promote cellular proliferation. Recent advancements in biomaterials have suggested whey protein isolate (WPI) hydrogel as a potential candidate to act as a scaffold with the capacity for drug delivery for bone regeneration. In this study, we investigate whey protein isolate hydrogels enhanced with the phytocannabinoid cannabidiol (CBD). The use of CBD in WPI hydrogels for bone regeneration is original. The results suggest that CBD was successfully incorporated into the hydrogels bound potentially through hydrophobic interactions formed between hydrophobic patches of the protein and the hydrophobic cannabinoid. The incorporation of CBD into the WPI hydrogels improved the mechanical strength of the hydrogels. The Young’s modulus was improved from 2700 kPa ± 117 kPa to 7100 kPa ± 97 kPa when compared to the WPI control, without plant-derived cannabinoids, to the WPI with the maximum CBD concentration. Furthermore, statistically significant differences for both Young’s modulus and compressive strength were observable between the WPI control and CBD hydrogel variables. The release of CBD from the WPI hydrogels was confirmed with the results suggesting a maximum release of 20 μM over the 5-day period. Furthermore, the hydrogels supported the proliferation and synthesis of collagen and calcium, as well as the alkaline phosphatase activity of MC3T3-E1 pre-osteoblasts, which demonstrates the potential of WPI/CBD hydrogels as a biomaterial for osseous tissue regeneration.

## 1. Introduction

Osteoporosis and the resulting breaks and fractures are the consequence of structural deterioration of the bone and low bone mass density [1]. Although bone has regenerative qualities, with age the regenerative properties diminish and fractures and breaks fail to heal correctly. Currently, the number of individuals aged over 60 is approximately 1 billion. This number is expected to rise to 1.4 billion by 2030 and 2.1 billion by 2050 [2]. This increase in aging will impose further strain on global healthcare services due to incidents stemming from age-related chronic pathologies such as osteoporosis. In the United Kingdom alone the annual cost to the National Health Service is expected to reach GBP 2.2 billion by 2025 [3].

Currently, the gold standard treatments for osteo-related pathologies are autogenous tissue grafts or, in more extreme cases, sectional implants such as hip replacements [4]. However, these procedures present their own limitations with non-union of the graft to the osteo site being the dominating limiting factor [5]. To address this problem, several synthetic and natural materials, alone or in combination, have been developed as scaffolds for tissue engineering (TE), a viable alternative to traditional grafting methods. However, current scaffolds have limitations [6]. Some materials fail structurally and present poor mechanical integrity or fail to distribute weight correctly, whilst some materials prove to be cytotoxic, non-osteoconductive, non-osteoinductive, or unable to promote osseointegration [7]. A further limiting factor is the inability of scaffolds to deliver hydrophobic molecules to the graft site. The inability to deliver hydrophobic drugs creates a barrier to potential treatments that could potentially reduce the chance of implant rejection by inhibiting infection and inflammation, whilst promoting cellular proliferation and differentiation, allowing for the formation of new bone [8]. Therefore, there remains a requirement for bone scaffolds with the potential to deliver hydrophobic molecules.

**Figure 1:**
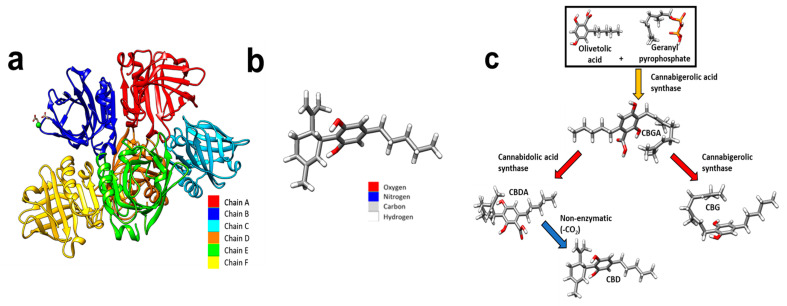
(**a**) Coloured depiction of the chains of beta-lactoglobulin. Beta-lactoglobulin consists of 6 chains, A–F. A—red, B—blue, C—cyan, D—orange, E—green, and F—yellow. (**b**) A schematic of a CBD molecule. (**c**) A schematic demonstrating the biosynthesis of CBD. Cannabigerolic acid (CBGA) is produced by interaction of olivetolic acid and geranyl pyrophosphate. CBGA is degraded into CBDA. CBDA is further degraded into CBD. Molecular graphics were made and analyses were performed with University of California, San Fransico (UCSF) Chimera [17]. The beta-lactoglobulin molecular structure was sourced from PubMed and the glutamic acid molecule was sourced from PubChem.

Recently, dairy-derived, whey protein isolate (WPI) has demonstrated the potential to be loaded with hydrophobic molecules. Derived as a waste product from the dairy industry, WPI consists of the purified proteins of whey with β-lactoglobulin as the main constituent (75%), Figure 1 [9]. The recent evaluation of WPI hydrogels suggests that WPI is a suitable scaffold for tissue engineering. The potential of WPI stems from its ability to form hydrogels through a mechanism of heat- or pressure-induced protein dissociation and denaturation [10,11]. The formed hydrogels are easily sterilisable and non-toxic during degradation [12]. Advantageously, WPI hydrogel has demonstrated the ability to load hydrophobic molecules. For instance, a previous report [13] described the synthesis of WPI hydrogels enriched with the hydrophobic drug phloroglucinol. The subsequent WPI–phloroglucinol hydrogels promoted the growth of human dental pulp stem cells and the production of collagen. Furthermore, inorganic phases or polymeric materials such as bioactive glass, aragonite, and poly-γ-gamma glutamic acid have been incorporated into WPI hydrogels; the results have demonstrated the potential of WPI hydrogels in a bone scaffolding role by supporting the proliferation of MG-63 cells [14,15,16].

CBD (Figure 1b) is a phytocannabinoid synthesised from cannabigerolic acid (CBGA), Figure 1c. CBD affects biological processes by acting as a ligand acting on cellular transmembrane receptors. CBD is a non-competitive cannabinoid receptor 1 (CB1R) and cannabinoid receptor 2 (CB2R) receptor antagonist with further notable ligand–receptor interactions with G protein coupled receptor 55 (GRP55), transient receptor potential cation channel subfamily V (TRPV), and peroxisome proliferator-activated receptors (PPARs) [18]. CB1 and CB2 receptors are ubiquitous in osteocytes, with the CB2 receptor being the most abundant, and the presence of GRPs and TRPV receptors also being recognised [19]. Furthermore, the presence of CB1 and CB2 receptors has been evidenced to have a positive effect on bone metabolism [20]. The role of CB2R has been suggested to be as an inducer of matrix formation [21]. In vitro analysis has also suggested a positive role in bone regeneration. For instance, Idris et al. [22] demonstrated an increase in osteoblasts in vivo and inhibition of osteoclasts, in vitro with CBD, achieved through CB1R and CB2R antagonism. Similarly, Apostu et al. [23] demonstrated increased osteoblast functioning whilst inhibiting osteoclasts. However, the mechanism suggested was through GPR55 antagonism. Similarly, and additionally, CBD promoted the osteogenic differentiation of bone marrow mesenchymal stem cells by p38 MAPK signalling pathway activation [24].

Microbial infection is often recorded as a causative agent in implant failure. CBD has demonstrated the inhibition of pathogenic microbial species, limiting the potential chance of microbial infections, increasing the chance of implant success [25,26]. Further mitigation of implant rejection is presented through the potential for CBD to reduce excess inflammation, limiting the chance of implant rejection. The anti-inflammatory properties of CBD have been established with [27] suggesting nuclear factor-κβ (NF-κβ) transcription inhibition as the main mechanism. Additionally, ref. [28] demonstrated the anti-inflammatory activity of CBD, suggesting a 50–65% decrease in neutrophil recruitment.

Therefore, we took the novel approach of combining the potential of WPI as a delivery vehicle for hydrophobic substances with the bone regenerative potential of hydrophobic CBD, synthesising WPI/CBD hydrogels. The investigation sought to evaluate any potential for WPI/CBD in bone regenerative medicine and to add to the understanding of WPI to deliver hydrophobic small molecules to treat chronic pathologies. The potential of the WPI/CBD hydrogel was analysed by immersion in a pH 7 solution, a pH consistent with an osteogenic environment, and evaluation of the hydrogel swelling characteristics. Second, mechanical compression testing was conducted to ascertain any potential influence on the Young’s modulus of the WPI hydrogels by the addition of CBD. Release profiling was conducted to analyse the WPI potential for CBD release from the hydrogels in a pH 7 environment. Finally, MC3T3-E1 pre-osteoblasts were utilised to analyse the potential of WPI/CBD hydrogels to promote cellular differentiation and cellular functioning.

## 2. Results and Discussion

### 2.1. Swelling

**Figure 2:**
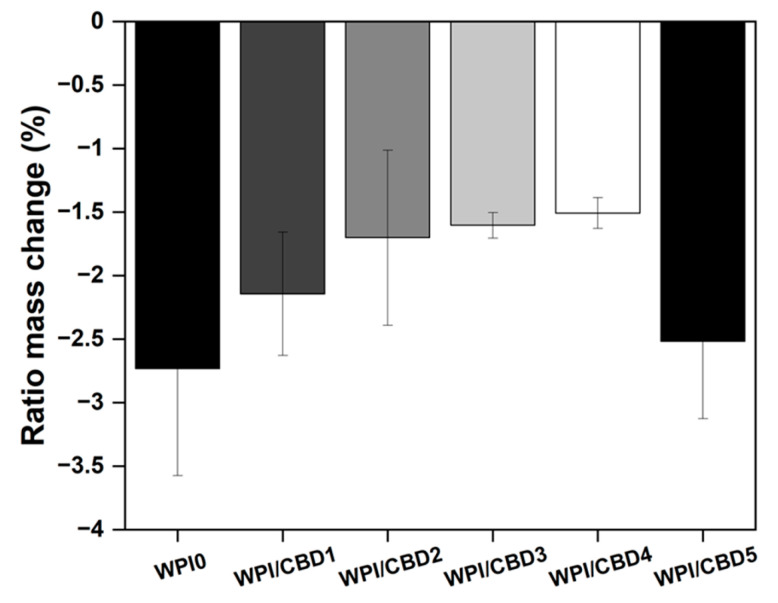
The results of a polymer swelling assay in pH 7 conditions presented as ratio mass change (%) of the WPI/CBD hydrogels (n = 10). No statistical significance was observed between the WPI/CBD hydrogel variables and the WPI hydrogel control with no CBD.

A swelling assay was performed to ascertain the swelling potential of the hydrogels in an osteogenic environment, with an optimal pH of 7.4, the results of which can be observed in Figure 2. Although some linear concentration dependence can be observed between WPI/CBD hydrogels and the WPI control samples, the effects fail to produce statistical significance. However, given the closeness to statistical significance it would be expected that only a minor further increase in concentration of either cannabinoid would yield statistical significance.

All hydrogels failed to swell during the assay. In fact, the hydrogels lost mass, resulting in a negative ratio mass change (%). The mass loss observed was likely the result of degradation of the hydrogels at pH 7. However, the addition of CBD exhibited an observable effect on the hydrogels. The effect was that an increase in CBD concentration had a positive effect on the degradation of the WPI hydrogels, when compared to the WPI control. The positive effect was demonstrated by a reduction in the mass loss. For instance, CBD improved mass loss linearly from a swelling mass ratio of −2.73 to swelling mass ratios of −2.14, −1.70, −1.60, and −1.51 for WPI/CBD1, WPI/CBD2, WPI/CBD3, and WPI/CBD4, respectively. However, the result for WPI/CBD5 did not follow the trend and it lost more mass. The reasons for this remain unclear, but it may be due to imperfections in the hydrogels resulting from the manufacturing process. For instance, bubbles can form in the hydrogel; these have been described in previous publications on WPI hydrogels [13,14,15]. It is unclear if these are due to residual dissolved air or formation of steam during sterilisation by autoclaving; in the absence of further data, this discussion must remain speculative. It is not inconceivable that the formation of bubbles may weaken the hydrogels’ structural integrity and promote the development of cracks and fractures.

WPI hydrogels incorporate hydrophobic molecules through hydrophobic interactions because of the exposure of hydrophobic regions and hydrophobic amino acids during the denaturing process [29]. Therefore, any change in ratio mass change (%) could potentially be attributed to the structural changes due to hydrophobic interactions between hydrophobic CBD and the WPI proteins. Therefore, it is potentially possible that even with the low concentration the results are due to the underlying hydrophobic interactions. However, due to the low concentrations utilized, further analysis would have to be undertaken to determine if the previous statement is factual. Additionally, the results of the WPI control align with WPI controls taken from previous investigations such as the aforementioned Baines et al. [16].

### 2.2. Mechanical Testing

**Figure 3:**
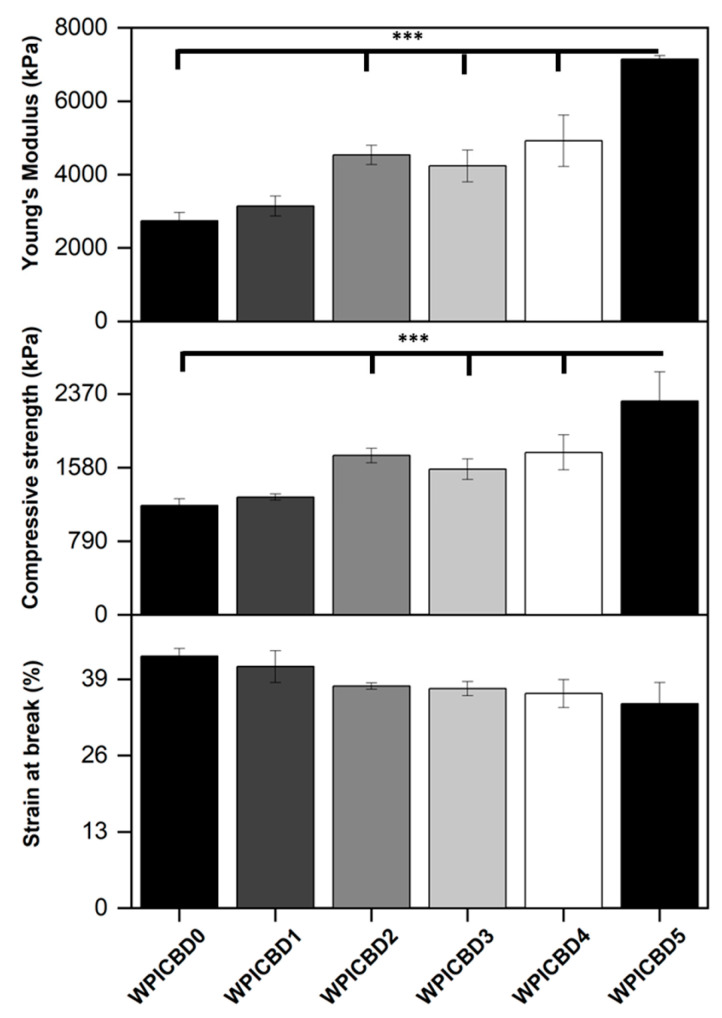
The results for the compression analysis of the WPI/CBD hydrogels. The results for Young’s modulus (kPa), compressive strength (kPa), and strain at break (%) are depicted. Each bar represents the mean ± SD of n = 10 (*** *p* < 0.001; compared to the WPI control).

Osteogenic scaffolding requires the potential to withstand loading. A limitation to WPI hydrogels as bone scaffolding is a lack of load-bearing potential. Therefore, mechanical assays were conducted to ascertain whether the addition of cannabinoids had any effect on the load-bearing potential of WPI hydrogels. The results of the assay can be observed in Figure 3. The WPI/CBD hydrogels presented some effect on the relative strength of the WPI samples as demonstrated by the results for Young’s modulus, compressive strength, and strain at break. There are some anomalies in the data, likely the result of imperfections in the hydrogels. However, an increase in cannabinoid concentration generally increased both the Young’s modulus and compressive strength of the samples containing cannabinoids. For example, the WPI0 control demonstrated a Young’s modulus of 2736 kPa compared to a result for the WPI/CBD5 hydrogel of 7146 kPa. Additionally, the data suggested statistically significant differences for all WPI/CBD variables apart from WPI/CBD1 when compared to the WPI control variable. Furthermore, the results gained from the WPI control variable are consistent with previous works, aligning with the aforementioned [13,14], respectively.

The reasons for the increases in Young’s modulus and compressive strength due to the addition of CBD remain unclear. It is conceivable that hydrophobic interactions between CBD and β-lactoglobulin may occur. During the gelation process, it is believed that β-lactoglobulin molecules unfold and expose hydrophobic regions to which hydrophobic molecules can bind, as shown previously [13]. Possibly, hydrophobic CBD may act as a crosslinker by forming hydrophobic bridges between hydrophobic regions of β-lactoglobulin molecules, acting as a crosslinker. This would explain the trend whereby increasing the CBD concentration leads to improved mechanical properties. However, in the absence of further evidence, this discussion must remain speculative.

### 2.3. Release Profiling

#### 2.3.1. Standardisation Process

**Figure 4:**
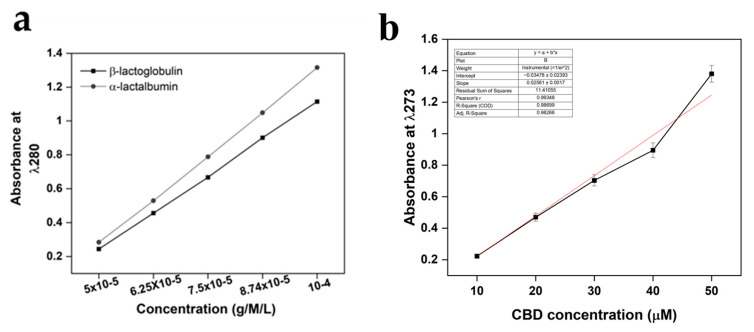
UV-Vis method standardisation calibration curves for both (**a**) β-lactoglobulin and α-lactalbumin at 280 nm, and (**b**) CBD calibration curves. For β-lactoglobulin and α-lactalbumin the region of interest was 280 nm, whereas the region of interest for CBD was 273 nm. The concentration units were g/M/L for both β-lactoglobulin and α-lactalbumin and μΜ for CBD. Each bar represents the mean ± SD of n = 15.

Amino acids found in β-lactoglobulin and α-lactalbumin demonstrate absorbance at 280 nm, resulting mainly from the aromatic portion associated with amino acids tryptophan and tyrosine. CBD has been demonstrated to present absorbance in the same region with a maximum absorbance peak at 273 nm. Therefore, a standardisation assay determined if small-scale differences could be observed in the aromatic-associated region. The results, observable in Figure 4, demonstrated a linear increase in concentration related to absorbance as would be expected. Moreover, the results demonstrated an expected difference in absorbance at each concentration between the β-lactoglobulin and α-lactalbumin variables, with β-lactoglobulin presenting less absorbance at each concentration level. No further statistical analysis was performed, other than standard error, due to the minor percentile differences between the proteins, meaning the samples were unlikely to yield any statistically significant differences (n = 15).

#### 2.3.2. CBD Calibration

**Figure 5:**
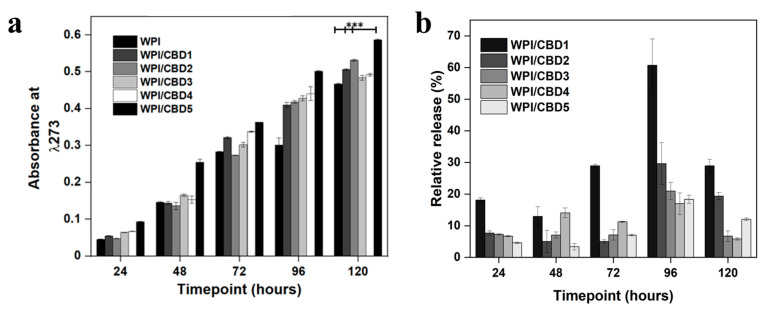
(**a**), representative CBD release data at each timepoint. The chart maps the release of CBD over 120 h. WPI has no CBD. The WPI/CBD1 samples contain 10 μΜ CBD. The WPI/CBD2 samples have 20 μM CBD. The WPI/CBD3 samples contain 30 μM CBD. The WPI/CBD4 samples have 40 μΜ CBD. The WPI/CBD5 samples have 50 μΜ CBD. The absorbance was analysed at 273 nm. Each bar represents the mean ± SD of n = 15 (*** *p* < 0.001; compared to the WPI control). (**b**), relative release percentage of CBD.

The results of the CBD calibration curve performed with UV-Vis can be observed in both Figure 5 and Table 1. The results provided a basis for the analysis for understanding the release of CBD for the WPI/CBD hydrogels. The results demonstrated a concentration-dependent linearity with each increasing CBD concentration. However, 50 μM deviated from the linear trend, resulting in higher-than-expected absorbance, potentially the result of background interference.

#### 2.3.3. CBD Release at pH 7.4

The release of CBD was monitored at a pH consistent with an osteogenic environment, the results of which can be observed in Figure 5a. The results demonstrated an increase in absorbance for the CBD concentrations WPI/CBD1, WPI/CBD2, WPI/CBD3, WPI/CBD4, and WPI/CBD5 with statistical significance shown for WPI/CBD1, WPI/CBD2, and WPI/CBD5 when compared to the WPI control sample (*p* < 0.05). Both WPI/CBD2 and WPI/CBD4 present anomalous results likely due in part to sample imperfections. The result at 120 h for the WPI0 control was subtracted from the maximum WPI/CBD result and can be cross-referenced with Table 1. The result suggests a 0.49 cm^−1^ increase in absorbance between the WPI0 control and the WPI/CBD, resulting in circa 20 μM. The absorbance data suggested 120 h as the maximum release was at 120 h. However, when the percentage of relative release was calculated (Figure 5b) the maximum release of CBD was at 96 h. When compared with the literature, the amount of CBD released in this study could potentially promote the osteogenic differentiation of mesenchymal stem cells, as previously mentioned in [24]. Additionally, CBD concentrations between 10 μM and 30 μM have been shown to inhibit osteoclast differentiation, demonstrating the effect of low concentrations of CBD on bone tissue remodelling [30]. Furthermore, CBD has exhibited antimicrobial activity at concentrations as low as 10 μM, suggesting the potential of the WPI/CBD hydrogel to prevent infection.

Previously, CBD release has been analysed by [31]. The data from [31] suggested that the maximum release of CBD from a transdermal patch was from 39–70 h. However, the WPI hydrogel system analysed here increased maximum potential release time to 96 h, which in part agrees with [32] who suggested that CBD release was sustained in vivo when released from a nanochannel delivery system. However, due to the protein nature of the WPI hydrogels—the hydrophobic binding mechanism underlying the formation of the WPI/CBD—the full release of CBD would be relevant to the degradation of the hydrogels and would only be complete upon the full degradation of the hydrogels.

### 2.4. Biocompatibility and Osteogenic Capacity of the Scaffolds

**Figure 6:**
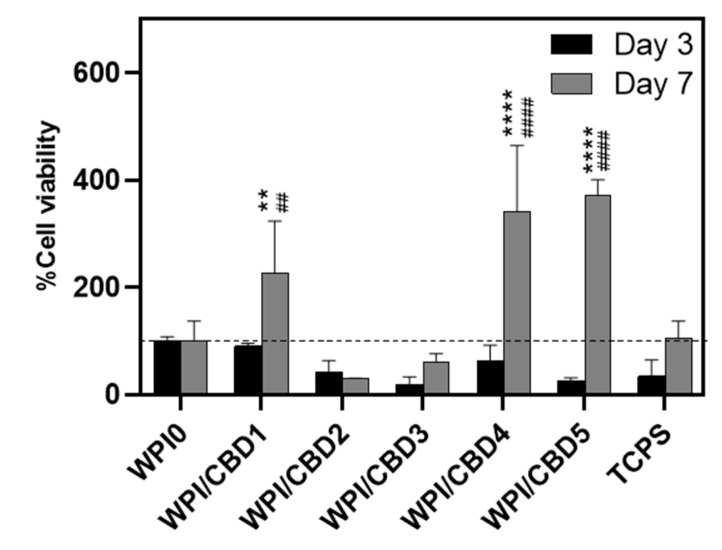
The results of % cell viability and proliferation of MC3T3-E1 pre-osteoblasts seeded on WPI/CBD hydrogels relative to WPI0 (set as 100%). TCPS is tissue-culture-treated polystyrene and serves as control. Absorbance readings at 570/600 nm were taken at days 3 and 7. Each bar represents the mean ± SD of n = 6 (** *p* < 0.01, **** *p* < 0.0001; compared to the WPI control) (^##^ *p* < 0.01, ^####^ *p* < 0.0001; compared to the TCPS control).

**Figure 7:**
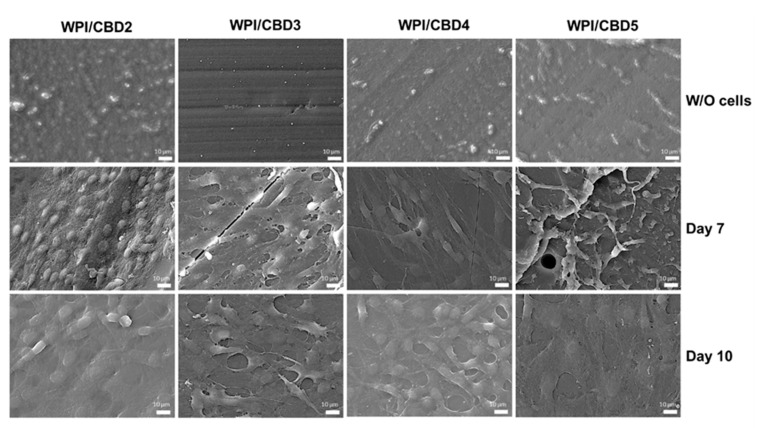
SEM images demonstrating the cellular morphology of MC3T3-E1 pre-osteoblasts on WPI/CBD hydrogels. Images were taken at 7 and 10 days of culture (**middle** and **lower panels**). The upper panel shows unseeded hydrogels (without cells) to facilitate comparison. Scale bars represent 10 μm.

In vitro analysis was conducted to determine the biocompatibility of the WPI/CBD hydrogel samples with MC3T3-E1 pre-osteoblasts. A PrestoBlue^TM^ cell viability assay was utilised and cytocompatibility was established from 3–14 days for WPI/CBD hydrogels. At the 7-day timepoint the WPI/CBD4 and WPI/CBD5 presented a significant difference in cellular proliferation when compared to the WPI0 control variable (*p* < 0.05). At day 14, all WPI/CBD hydrogels had demonstrated the potential for cellular proliferation, as demonstrated in Figure 6. Additionally, MC3T3-E1 proliferation is observable in SEM images in Figure 7. The hydrogels WPI/CBD1, WPI/CBD2, WPI/CBD4, and WPI/CBD5 presented higher absorbance readings than the WPI0 control but failed to produce statistically significant data. Additionally, the results support the release of CBD from the hydrogels, demonstrated through biological activity.

**Figure 8:**
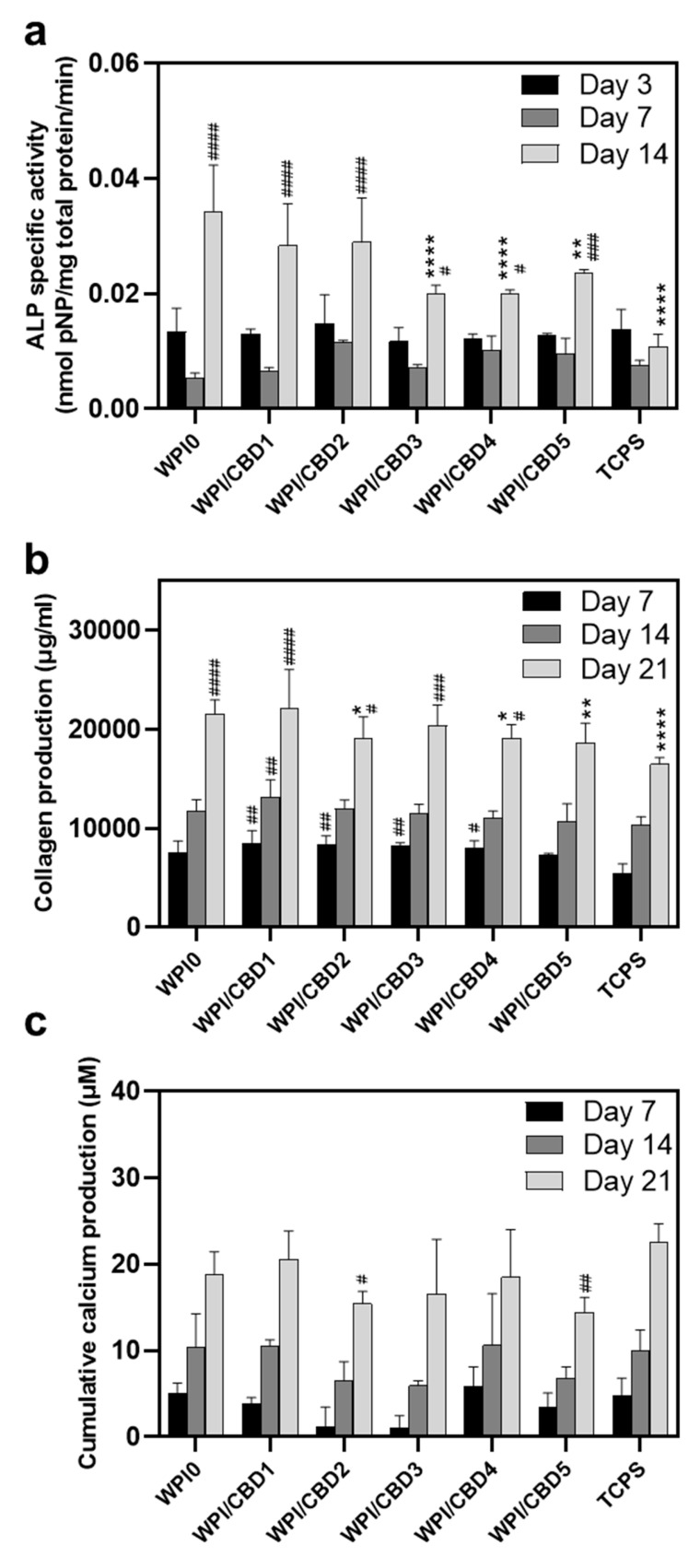
Assessment of the osteogenic potential of MC3T3-E1 pre-osteoblastic cells cultured on WPI/CBD hydrogels; (**a**) the expression of normalised ALP-specific activity on days 3, 7, and 14, (**b**) collagen production, and (**c**) calcium production by pre-osteoblasts on days 7, 14, and 21. Each bar represents the mean ± SD of n = 6 (* *p* < 0.05, ** *p* < 0.01, **** *p* < 0.0001; compared to the WPI0 control) (^#^ *p* < 0.05, ^##^ *p* < 0.01, ^###^ *p* < 0.001, ^####^ *p* < 0.0001; compared to the TCPS control), while the absence of asterisks in the graph of calcium production (**c**) signifies statistically non-significant differences when compared to the WPI0 control.

**Figure 9:**
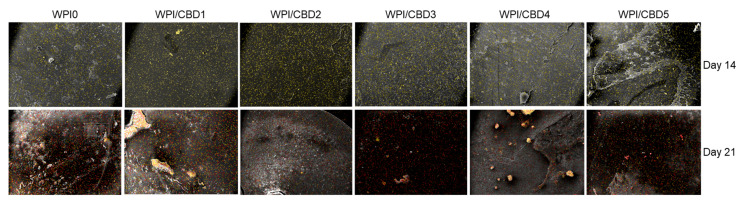
A visual representation of the determination of the mineralisation through atomic-resolution mapping of calcium and phosphorus via EDS analysis. The top row depicts day 14 and the bottom row depicts day 21. Calcium is highlighted in yellow, while phosphorus is highlighted in red.

Further analysis was conducted on the WPI/CBD variables. The analysis sought to establish the metabolic functioning of the MC3T3-E1 pre-osteoblasts. The results of this are observable in Figure 8. The analysis investigated the concentrations of three markers synonymous with osteogenic differentiation: two protein-based, namely ALP activity and collagen secretion, and one mineral-based, namely calcium production. ALP has been demonstrated to catalyse calcium mineralization, synthesising new bone and negatively regulating an inhibitor of mineralisation, extracellular pyrophosphate, while collagen is one of the main constituent proteins of the extracellular matrix, and calcium mineralisation is indicative of new bone formation, making all three markers for bone regeneration [33,34,35]. The results for each marker are displayed in Figure 8. Their activity was observable in the cells seeded on the WPI/CBD hydrogels. Additionally, EDS analysis in Figure 9 suggests deposits of both calcium and phosphorus, markers of cellular mineralisation. Future work could involve measurement of Ca and P amounts to further characterise the mineral phase.

It is important to note that all groups, including TCPS and WPI/CBD hydrogels, were cultured under identical osteogenic conditions; the higher calcium production observed in the TCPS control may be attributed to its stiff nature, which may promote late-stage osteogenic differentiation and mineralisation, as previously reported for the effect of the substrate stiffness on cell responses [36]. In contrast, the softer, three-dimensional WPI/CBD hydrogels support early osteogenic activity, while potentially modulating or delaying terminal calcium deposition [37].

These results suggest that CBD retains its biological activity to a certain extent after autoclaving. The fact that CBD is detected (Figure 5) indicates that CBD is not destroyed by autoclaving. Previous work has demonstrated that phloroglucinol, the phenolic subunit of phlorotannins, polyphenols occurring in brown seaweed, retains biological activity after incorporation in WPI hydrogels and sterilisation by autoclaving [13]. Further work is necessary to elucidate the exact effect of autoclaving on biological activity.

The positive effect on cellular functioning established in this study supports previous investigations which suggest that CBD has a potential role in osseous regeneration. As suggested in the Introduction, CBD enables biological responses by acting on cellular receptors, most notably CB1R and CB2R. The role of CB1R and CB2R in osseous functioning has been well established. The presence of CB1 and CB2 receptors in osteoblasts has been evidenced to be abundant, as previously mentioned in [19]. Notably, it was suggested by [38] that, once acted on, the receptors have a positive metabolic effect. Similarly, ref. [39] suggested that CBD treatment enhanced bone healing. Furthermore, CBD acting as a GPR55 antagonist increased the functioning of osteoblasts as demonstrated by [40]. CBR1 and CBR2 knockout mouse models produce contrasting results with a decrease in bone mass being observed when CB1R and CB2R are deleted. However, background age and sex introduce variability into the results. For instance, CBR1 deletion in a female C57Bl/6 mouse presented a decrease in bone density. Similarly, when CBR1 was inhibited in a mouse model with the same background the results supported the previous investigation. However, CD1 male and female mice with a CD1 background demonstrated an increase in bone density at three months [41]. Our findings support that CBD can promote osseous regeneration. However, the pathway is yet to be determined.

## 3. Conclusions and Outlook

This preliminary investigation sought to manufacture WPI hydrogels with incorporated CBD and evaluate their potential use as biomaterials or scaffolds for bone regeneration. Numerous conclusions can be taken from the study: the successful incorporation of CBD into the WPI hydrogel; a difference in both the swelling and mechanical behaviour of the WPI hydrogels with the addition of CBD influencing their physical properties is observed, as demonstrated by the significant differences in the mechanical analysis.

Release investigations demonstrated the release of CBD from the hydrogel. Significant differences were observed between WPI/CBD0 and the WPI/CBD. WPI/CBD1, WPI/CBD2, and WPI/CBD5 demonstrated significant differences when compared with the WPI0 control. Evidence for the release of CBD was further supported by the cellular viability analysis. Specifically, CBD retained its biological activity post-autoclaving, as indicated by its ability to promote pre-osteoblastic cell proliferation (Figure 6). However, CBD did not consistently enhance osteogenic differentiation markers, suggesting that its primary effect may lie in early-stage cell activation rather than later-stage differentiation. Additionally, The WPI/CBD hydrogels supported the cellular functioning of pre-osteoblasts. Therefore, the potential of WPI to encapsulate and deliver hydrophobic molecules and the potential of WPI/CBD hydrogel in bone regeneration were demonstrated.

Further work should focus on several questions emerging from this study, including a study of the effect of the sterilisation technique on the biological activity of CBD; an elucidation of the mechanism by which CBD reinforces WPI hydrogels mechanically; as well as a more detailed examination of the mineral formed, perhaps by use of Raman spectroscopy. Optimisation of the WPI/CBD hydrogels may include expanding the range of CBD concentrations or WPI concentrations or the use of different sterilisation methods (e.g., gamma sterilisation or electron-beam crosslinking and simultaneous sterilisation), use of primary osteoblasts or mesenchymal stem cells, and elucidation of the pathway by which CBD exerts is biological effect.

## 4. Materials and Methods

### 4.1. Materials

WPI (>97% protein, of which >75% β-lactoglobulin) was obtained from Davisco, Savage, MN, USA. CBD was obtained from Tocris, Bristol, UK. Unless stated otherwise, cell culture reagents and consumables were obtained from PAN-Biotech, Aidenbach, Germany.

### 4.2. WPI CBD Hydrogel Formation

WPI–cannabinoid hydrogels were formed under heat-induced disassociation. The hydrogels were formed with a concentration of 40% WPI (*w*/*v*) with ultrapure H_2_O. The addition of cannabinoid CBD created a WPI–cannabinoid hydrogel with concentrations of 10 µM, 20 µM, 30 µM, 40 µM, and 50 µM. Homogenisation was achieved firstly by vortexing the solution before full homogenisation was achieved utilising an IKA Loopster for 24 h. The induction of gelation was achieved by placing 1 g samples in a 70 °C water bath for 5 min. Sterilisation was achieved with an autoclave at 126 °C and 1.5 bar of pressure for 15 min (Table 2).

### 4.3. Swelling

To determine both the effect of the structural behaviour of the hydrogels with the incorporation of the cannabinoids and the effect of the neutral pH of the osteogenic environment on the hydrogels, swelling assays were performed. The method was as follows: WPI–cannabinoid hydrogel samples with a height of 10 mm, a diameter of 8 mm, and a mass of 1 g were introduced to a 5 mL pH 7.4 solution, namely phosphate-buffered saline (PBS). The initial mass of the hydrogels was measured before the samples were incubated for 1 week. Post-incubation the final mass was measured, and the swelling mass ratio was calculated using the formula below where the swelling percentage S% is calculated from the wet mass Mw and the dry mass Md.

S% = (Mw − Md)/Md × 100%

### 4.4. Mechanical Analysis

Any potential scaffold requires sufficient compressive strength. Therefore, it was important to analyse any potential changes the addition of cannabinoids cause to the compressive potential of WPI hydrogels. Previously, WPI hydrogels have presented less than desirable compressive potential. Therefore, compression analysis was conducted to ascertain if the addition of cannabinoids had any influence on WPI hydrogel compressive abilities. Compression testing was achieved using an Instron 3345 (United States of America). WPI hydrogel samples with additional CBD concentrations of 10 µM, 20 µM, 30 µM, 40 µM, and 50 µM were developed with a height of 10 mm and a radius of 4 mm. The rate of compression was 2 mm per min.

Youngs modulus (Ε) was calculated as

Ε = σ/∈

where σ denotes stress and ε denotes strain.

Compressive strength was calculated as

F = P/(πr2)

where P is the load at failure and A is the cross-sectional area.

Strain at break was calculated as

∈ =∆L/L × 100

where ΔL is the difference between the initial length and the final length and L is the initial length.

### 4.5. Release Profiling

#### 4.5.1. Standardisation Assay

##### β-Lactoglobulin and α-Lactalbumin Standardisation

The standardisation assay was conducted to provide preliminary validation in the methodology. β-lactoglobulin and α-lactalbumin were chosen as the molecules for standardisation for two key reasons. Firstly, β-lactoglobulin and α-lactalbumin are the two main protein components of WPI. Secondly, β-lactoglobulin and α-lactalbumin present differing amounts of tryptophan (Trp) and tyrosine (Tyr) residues with percentages of 1.1% and 2.8% for Trp and 2.2 and 2.8 for Tyr, respectively. Due to the aromatic portion of the respective molecules, both Try and Trp exhibit absorbance at 280 nm. CBD possesses an aromatic portion and displays maximum absorbance at 273 nm.

Both β-lactoglobulin and α-lactalbumin solutions were formed with max concentrations of 10^−4^ g/M/L. The solutions were serially diluted and analysed on a UV-Vis NanoDrop (ThermoFisher). The method utilised in the investigation was adapted from a previously described method by [42].

##### CBD Calibration

CBD solutions were 50 μM in 95% ethanol. The solution was serially diluted and analysed as per the β-lactoglobulin and α-lactalbumin standardisation, obtaining data for absorbance at 273 nm.

#### 4.5.2. CBD Release Profiling

To enable CBD to act with its known biological activity, the release from the WPI hydrogels is advantageous. Here, 1 g WPI/CBD samples were introduced to 5 mL PBS. The samples were incubated at 37 °C. At 24 h timepoints, 100 µL was removed from each sample and replaced with 100 µL of fresh PBS for 5 days. The samples were analysed utilising UV-Vis spectroscopy with an emphasis on 273 nm, the known region of CBD (n = 15).

Percentage of relative release was calculated as

Relative release (%) = (CBD concentration at time (t)/Max CBD concentration × 100

### 4.6. Cell Culture and Viability

Cellular viability and behaviour assays were conducted utilising the mouse calvaria osteoblast precursor cell line (MC3T3-E1, DSMZ Braunschweig, Germany, ACC-210). The cells were cultured in alpha-MEM medium supplemented with 10% foetal bovine serum (FBS), 2 mM L-glutamine, 100 μg/mL penicillin/streptomycin, and 2.5 μg/mL amphotericin (Thermo Fisher Scientific, Waltham, MA, USA) and incubated in a 5% CO_2_ incubator at 37 °C (Heal Force, Shanghai, China). When the cells had reached confluence, they were detached with trypsin–0.25% ethylenediaminetetraacetic acid (EDTA) (Gibco, Thermo Fisher Scientific, Waltham, MA, USA) and were seeded onto WPI/CBD hydrogels previously sterilised through UV radiation for 10 min. Each concentration of hydrogel was treated with 25 × 10^3^ cells for the proliferation assay and 40 × 10^3^ cells per scaffold for the differentiation assays, suspended in 10 μL complete media. Additionally, 400 μL culture medium was seeded onto each hydrogel. L-ascorbic acid (50 μg/mL), β-glycerophosphate (10 mM), and dexamethasone (10 nM) (all provided by Sigma, Darmstadt, Germany) were added to the primary culture medium for differentiation assay purposes.

A PrestoBlue™ (Invitrogen Life Technologies, Waltham, MA, USA) viability assay was conducted to assess the cellular viability of the WPI/CBD hydrogels. The hydrogels were treated with MC3T3-E1 pre-osteoblastic cells (n = 6). The assay utilised a resazurin-based indicator, a methodology previously described by [43]. The resazurin-based indicator stains cells, resulting in a red product that can be analysed photometrically. At timepoints (days) 3 and 14, 40 μL of PrestoBlue™ reagent, diluted in alpha-MEM at a 1:10 ratio, was added to each well and incubated at 37 °C for 60 min. Post-incubation, 100 μL of the supernatant was transferred to a 96-well plate and absorbance analysed at 570 and 600 nm. The assay employed a Synergy HTX Multi-Mode Microplate Reader (BioTek, Bad Friedrichshall, Germany). After this, the cell-seeded scaffolds were rinsed twice with PBS, and their culture media were renewed.

### 4.7. Cell Adhesion and Morphology Evaluation via Scanning Electron Microscopy

Scanning electron microscopy (SEM) was used to examine cell attachment and morphology on the WPI/CBD hydrogels. The assay was conducted with a JEOL JSM-6390 LV scanning electron microscope (JEOL Ltd., Akishima, Japan), with an accelerating voltage of 20 kV. MC3T3-E1 pre-osteoblastic cells (25 × 10^3^ cells per sample) were seeded onto the hydrogels and incubated in a CO_2_ incubator at 37 °C for 7 days. The hydrogels were rinsed with PBS and fixed using a 4% *v*/*v* paraformaldehyde solution for 15 min. Fixed hydrogels were dehydrated with ethanol with concentrations ranging from 30% to 100% *v*/*v*. The hydrogels were dried in a critical point dryer (Baltec CPD 030, Bal-Tec AG, Pfäffikon, Switzerland) and gold coated with a 20 nm thick layer of gold using a sputter coater (Baltec SCD 050).

### 4.8. Alkaline Phosphatase (ALP) Activity

ALP has a vital role in bone mineralisation and therefore is a model biomarker to determine cellular functioning of pre-osteoblasts. ALP activity on was assessed over a 7-day period with timepoints on days 3 and 7. The hydrogels (n = 6) were prepared by washing in PBS and introduced to 350 μL lysis buffer. The lysis buffer was prepared according to the following formula: 0.1% Triton X-100 and 50 mM Tris-HCl and buffered to pH 10.5. Three freezing/thawing cycles were conducted with temperatures ranging from ambient to a maximum low of −20 °C. Upon completion of the third cycle, a 100 μL suspension was homogenised with 100 μL of 2 mg/mL p-nitrophenyl phosphate (pNPP, Sigma, Darmstadt, Germany). The solution was diluted further in a buffer containing 50 mM Tris-HCl and 2 mM MgCl_2_ before incubation at 37 °C for 1 h. Colour change was measured at 405 nm with a spectrophotometer. The enzymatic activity was calculated using the formula [units = nmol p-nitrophenol/min]. Bradford (AppliChem GmbH, Darmstadt, Germany) protein concentration assay was utilised to determine cellular protein concentrations of the lysates.

### 4.9. Determination of the Produced Extracellular Collagen

Sirius Red dye assay (Direct red 80, Sigma-Aldrich, St. Louis, MO, USA) was conducted to determine the concentration of collagen secreted by MC3T3-E1 pre-osteoblastic cells (n = 6). The analysis was conducted over a 21-day period with the supernatants being collected every 3 days. The supernatants were diluted 1:4 with deionised water, producing a total volume of 100 µL. The solution was homogenised with 1 mL of 0.1% Sirius Red dye in acetic acid and incubated for 30 min in ambient conditions. Post-incubation the samples were centrifuged at 15,000× *g* for 15 min. The resulting pellets were washed with 0.1 N acetic acid removing any excess dye. The samples were dissolved in 500 µL of 0.5 N NaOH. The absorbance was measured at 530 nm using a Synergy HTX plate reader (Agilent Technologies, Santa Clara, CA, USA). The acquired data were cross-referenced with a collagen type 1 calibration curve.

### 4.10. Calcium Concentration Determination

A method using O-cresol phthalein complexone (CPC) (Biolabo, Maizy, France) was employed to quantitatively analyse calcium in supernatants (n = 6) according to a methodology previously described by [44]. Calcium deposition is an indicator of osteogenesis, having a significant role in the development of the bone extracellular matrix. The investigation was conducted over a 21-day period with the supernatants being collected every 3 days. From each sample, 10 μL of culture medium was diluted in 100 μL of calcium buffer with an additional 100 μL of CPC calcium dye. The solutions were analysed with absorbance calculated at 550 nm.

### 4.11. Statistical Analysis

Statistical analysis was carried out using an ANOVA *t*-test in GraphPad Prism version 8 software to assess the significance of differences among various scaffold compositions and the control at different experimental time periods. A *p*-value (*) less than 0.05 was considered significant (* *p* < 0.05), ** *p* < 0.01, *** *p* < 0.001, **** *p* < 0.0001, and “ns” denoted a statistically non-significant difference compared to the WPI control scaffold at the corresponding timepoint.

## Figures and Tables

**Table 1:** The numerical data gained from the UV-Vis calibration curve of CBD.

CBD Concentration (μM)	Absorbance at 273 nm	SD ±
10	0.22	0.01
20	0.47	0.03
30	0.70	0.04
40	0.90	0.05
50	1.38	0.05

**Table 2:** Experimental concentrations of WPI/CBD hydrogels.

Sample Name	WPI %	Cannabinoid Concentration (µM)
WPI0	40	0
WPI/CBD1	40	10
WPI/CBD2	40	20
WPI/CBD3	40	30
WPI/CBD4	40	40
WPI/CBD5	40	50

## Data Availability

Data are contained within the article.
